# Barriers to Breast Cancer-Screening Adherence in Vulnerable Populations

**DOI:** 10.3390/cancers15030604

**Published:** 2023-01-18

**Authors:** Laura Ponce-Chazarri, Jose Antonio Ponce-Blandón, Palmira Immordino, Antonio Giordano, Fátima Morales

**Affiliations:** 1 Department of Preventive Medicine and Public Health, School of Medicine, University of Seville, 41009 Sevilla, Spain; 2 Spanish Red Cross Nursing College, University of Seville, 41009 Sevilla, Spain; 3 Department of Health Promotion, Mother and Child Care, Internal Medicine and Medical Specialties, University of Palermo, 90127 Palermo, Italy; 4 Sbarro Institute for Cancer Research and Molecular Medicine, Center for Biotechnology, College of Science and Technology, Temple University, Philadelphia, PA 19122, USA; 5 Department of Medical Biotechnology, University of Siena, 53100 Siena, Italy

**Keywords:** breast cancer screening, barriers, race, ethnicity, adherence

## Abstract

**Simple Summary:**

Breast cancer is the most prevalent cancer in the world. Early diagnosis can prevent cancer growth and therefore saving lives. Breast cancer screening through periodic mammography has been effective in decreasing mortality. However, adherence to screening does not meet the desired expectations in all population groups. The objective of this review is to identify the barriers that affect adherence to breast cancer–screening programs in an ethnically diverse group of women in order to propose public health measures to increase adherence. Although the dissemination of breast cancer–screening programs is still lacking in most of the vulnerable populations, we observed important favorable changes in those cases in which the population undergoes health education sessions, they are informed about cancer-screening programs or they seek medical attention. Therefore, implementing awareness campaigns focused on these populations should be promoted, as well as working on healthcare professional cultural competence to improve breast cancer–screening adherence worldwide.

**Abstract:**

Breast cancer screening through periodic mammography has been effective in decreasing mortality and reducing the impact of this disease. However, adherence to screening does not meet the desired expectations from all populations. The main objective of this review is to explore the barriers that affect adherence to breast cancer–screening programs in vulnerable populations according to race and/or ethnicity in order to propose measures to reduce the lack of adherence. We conducted a search of publications in the PubMed Central and Scopus databases. The eligible criteria for the articles were as follows: original quantitative studies appearing in SJR- and/or JCR-indexed journals from 2016 to 2021 in English or Spanish. Most of them present common barriers, such as race/ethnicity (47%), low socioeconomic (35.3%) and educational levels (29.4%), no family history of cancer and being single (29.4%), medical mistrust and a health information gap (23.5%), lack of private health insurance (17.6%) and not having annual health checks (17.6%). The target populations with the lowest adherence were Black, Asian, Hispanic and foreign women. Implementing awareness campaigns focused on these populations should be promoted, as well as working on diversity, cultural acceptance and respect with healthcare workers, in order to improve breast cancer–screening adherence worldwide.

## 1. Introduction

Breast cancer is the most prevalent type of cancer worldwide, with 2.2 million cases reported in 2020, making it the leading cause of death among women. Specifically, 685,000 deaths were recorded in 2020 because of this disease, according to the latest data by the World Health Organization (WHO). It is estimated that one in every 12 women will have breast cancer during her lifetime [1], with the highest incidence being in those of 45–65 years old, when hormonal changes occur in the pre- and post menopause period [2]. Although the average survival rate for this cancer at 5 years is 89.2% overall, the stage at which it is diagnosed has a great influence, varying from 98% to 24% survival depending on whether it is diagnosed at stage I or stage IV, respectively [3]. According to these considerations, and assuming the impact that breast cancer has in women’s lives, we can affirm that it is a public health problem worldwide. However, breast cancer mortality in high-income countries is decreasing thanks to research and improved treatments, together with the increased implementation of screening and early-diagnosis programs.

In Europe, a breast cancer–screening program consists of performing a mammography every 2 years on the asymptomatic women aged between 50 and 69 years old. Mammography is the most widely available test to diagnose breast cancer in asymptomatic and localized stages. In Spain, it has been shown that this screening modality reduced breast cancer mortality by 9% to 15% [4]. However, despite the proven effectiveness of this intervention, low levels of health literacy and inadequate knowledge about cancer screening, reported among women belonging to vulnerable populations, represent a serious concern. In fact, it is very common for these population groups to do not know what a cancer screening consists of, where accessing it or the possible consequences [5,6,7]. As a result, there are disparities in 5-year survival data of 90% in high-income countries versus 40–66% survival in low-income countries. The highest percentage of age-standardized deaths from breast cancer is in countries from Africa, where up to 50% of deaths from breast cancer occur in women aged under 50 years old [1].

In the United States (US), lack of medical insurance [5,6,7,8,9,10] appears as the main barrier to adherence or follow-up to cancer-screening programs for this disease in women belonging to certain population groups. Medical insurance financially protects the affected women from the multiple expenses that may arise from participation in this program or, even more, from the diagnosis of a suspected breast neoplasia. Some studies show a great lack of information in low-income populations, who admit to not having responded to the offer for cancer screening because they do not know what it consists of and are unaware of the disease risk factors, signs or symptoms [5,6,7,8,9,10]. Advani et al. insist on the need to improve the means of disseminating information to women, especially to those belonging to vulnerable groups [11]. The need for health education, particularly in vulnerable populations, promoting positive attitudes and behavioral changes among women would be effective in achieving better results in cancer-screening programs [10]. Other obstacles that appear in most of the studies are psychological barriers, mobility difficulties, language and cultural barriers, lack of time and/or the prioritization of other health issues [5,6,7,8,9,10,11,12].

The discomfort suffered by women in relation to being treated or examined by a male doctor is reported as a barrier in some studies [6,7,11,13]. Some of these women also reported that they would feel more comfortable being attended by female doctors, as well as an overall lack of confidence in the health system, based on personal experience and negative experiences reported in the media or through their own experiences [7]. In addition, feeling discriminated on the basis of their race and/or ethnicity also increases distrust in health systems. In an interview-based survey, Miller et al. [6] reported that Asian or Black women had been treated worse than white women in the United States (US).

On the other hand, a good doctor–patient relationship (DPR) is considered a positive factor for adherence, as it generates trust, comfort, and compliance with follow-up, which facilitate communication and participation in cancer-screening and early-detection programs [6,7].

Factors such as a previous history of breast cancer associated with participation in such screenings [5], a positive attitude toward the possible early diagnosis of the disease [5,9], family support [7,8,13] and being married [7,8] have been related to better adherence and follow-up. Being informed and advised by a health professional about the importance of this early cancer diagnosis has stimulated higher participation in breast cancer screening among women [7,11,13]. The abovementioned factors highlight the importance of keeping health professionals informed on the cultural, linguistic, and social differences in a target population for cancer screening, to be able to adapt and adopt customized prevention campaigns [11]. Situations such as a family history of breast cancer or other cancers, together with a personal history of early menarche, obesity, and other risk factors, do not seem to have shown evidence of greater adherence [5]. Age was shown to be a controversial factor as a variable for adherence to screening [8].

The WHO aims to achieve a 2.5% reduction in annual deaths from breast cancer, avoiding 25% of deaths by 2030. The success of screening programs is conditioned mainly by the rate of participation, so it is vital to encourage all populations to participate in them [14]. However, there are certain populations that could be at a disadvantage in properly participating in breast cancer screening. Hence, we review the psychosocial, socioeconomic, and cultural factors that interfere with adherence to breast cancer–screening programs in vulnerable populations owing to their race and/or ethnicity in order to obtain a global vision of participation and knowledge about the program, suggesting recommendations to reduce possible inequities.

## 2. Materials and Methods

PubMed Central and Scopus are two of the widest databases consulted by medical researchers, as they are comprehensive and facilitate researchers’ finding relevant and authoritative research. We selected both databases for our search. We chose the latest scientific reports on the topic for our research, including studies published in the past 5 years, from 1 September 2016, to 1 December 2021. The process followed for the selection of the articles was the same for both databases. The search strategy was performed using MeSH (medical subject headings) and DeCS (descriptors in health sciences) term-controlled language. The first step in developing the search strategy was to group a series of descriptors that made the results fit the main topic of the study. The Boolean operators AND, OR, parentheses and quotation marks were, then used to elaborate the following strategy: Breast Neoplasm AND (screening OR early detection of cancer) AND (ethnicity OR racial group) AND (social determinant of health OR social factor). The descriptor “Breast Neoplasm” was changed to “Breast Cancer” in the Scopus database because it offered a greater number of results. These strategies ended up with 305 publications that met the requirements: 181 studies in the PubMed Central database and 124 results in the Scopus database.

**Scheme 1:**
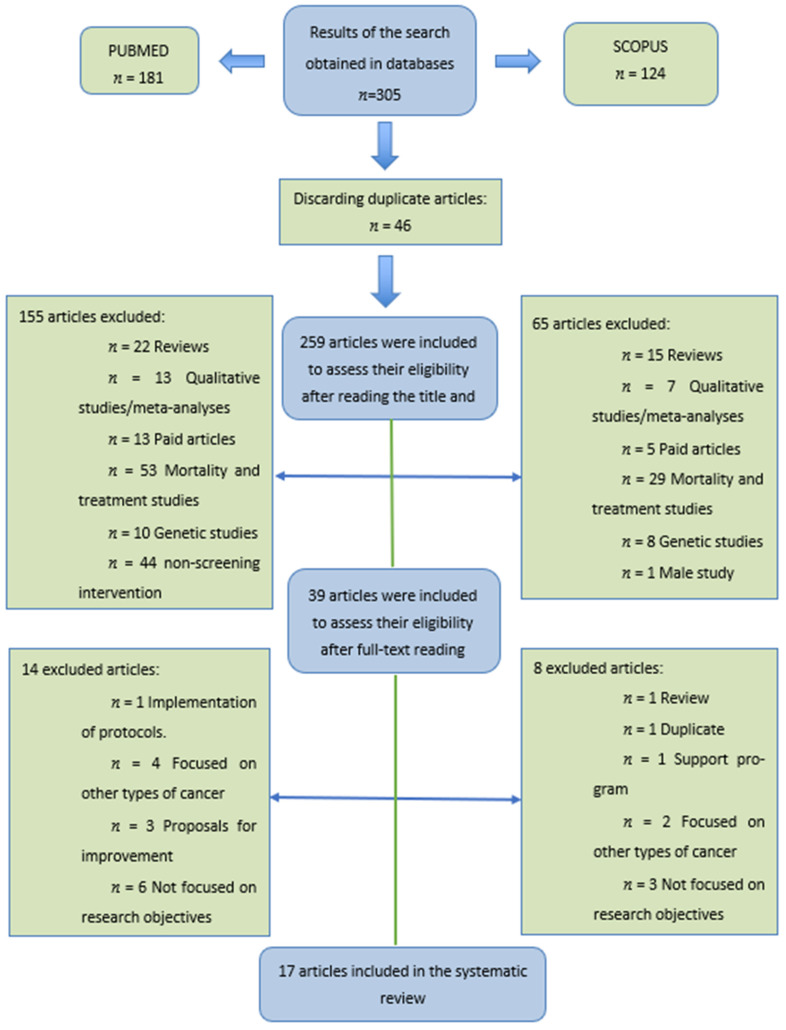
Flowchart of the article-selection process.

The eligible criteria included original articles with quantitative research of an observational or experimental design, that were written in English or Spanish, that were about breast cancer screening in women over 18 years old (inclusion criteria). We excluded studies that (i) were focused on predisposing genetic factors, (ii) were based on cancer therapy, (iii) analyzed breast cancer in terms of mortality and/or survival and (iv) were in books, conference abstracts, narrative review articles, meta-analyses, letters to editors or case studies (exclusion criteria). A researcher was responsible for the first search. However, when she hesitated on the inclusion or exclusion of some article for the research, she consulted with another researcher to make definitive decisions. To assess the risk of bias in the included studies, a third, independent researcher evaluated the proper inclusion of the selected papers in the review. The article-selection process is detailed in Scheme 1.

The quality of the articles was checked by selecting articles published in journals indexed in the Journal Citation Report (JCR) and/or Scimago Journal & Country Rank (SJR), including the impact factor of the year of publication. The articles were also evaluated using the critical-reading program Critical Appraisal Skills Programme España (CASPe). The questions included in this program analyze the internal validity of the study in terms of methodological adequacy and accuracy. The three main questions that this questionnaire aims to answer are as follows: (i) are the results valid, (ii) what are the results, and (iii) are they applicable in your setting? In total, 17 original publications were included in our research.

## 3. Results

The 17 studies included in our review are detailed in Table 1, along with their main results.

### 3.1. Adherence to Breast Cancer–Screening Programs

Levels of adherence to breast cancer-screening program are low in vulnerable populations, especially in women of Black or African American origin status [15,18,19,21,24]. Some common barriers that appear in most studies regarding nonadherence are (Table 2) not having an annual health checkup [22,26,28], belonging to a Black community [15,18,19,21,24], low level of education [15,19,25,27,31], low income and low socioeconomic status [16,18,19,23,24,27], lack of private health insurance [21,25,30] and the presence of medical mistrust and a health information gap [22,24,29,30]. Being single [17,26], being a foreigner [16,17,29] or not having a family/personal history of cancer [25,30,31] have also been related to lower adherence, in some studies.

### 3.2. Race and/or Ethnicity

**Figure 1:**
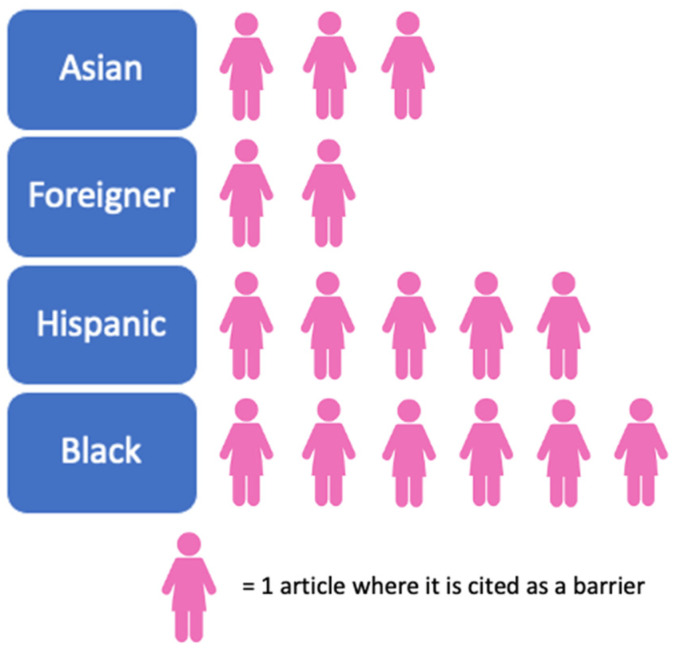
Barriers reported to race and/or ethnicity, according to number of articles.

A common factor related to a lower level of adherence to breast cancer screening is determined by the race and/or ethnicity of the women participating. There are multiple studies that have analyzed this barrier [15,18,19,21,24], which are reported in the 47% of the total articles researched (Table 1), which correlate race/ethnicity and country of birth as an impediment to the correct coverage of this screening program. The articles compare adherence in non-Hispanic white women with that in women of other ethnicities/races, as represented in Figure 1.

Black race/ethnicity is the one mentioned in the highest number of articles as a barrier to adherence (*n* = 6), followed by Hispanic (*n* = 5), Asian (*n =* 3) and foreign status (*n =* 2). If we focus on the data of non-Hispanic white women compared with Black women, the latter show a lower frequency of screening mammography [19], a higher probability of reporting barriers (odds ratio—OR: 1.30) [18] and higher rates of late diagnosis (OR: 1.56) [20]. In addition, the rate of discrimination was five times higher in Black and Hispanic women (11.2% and 11.3%) than in white women (2.2%) [21].

### 3.3. Socioeconomic Level, Lack of Resources and Lack of Private Health Insurance

**Figure 2:**
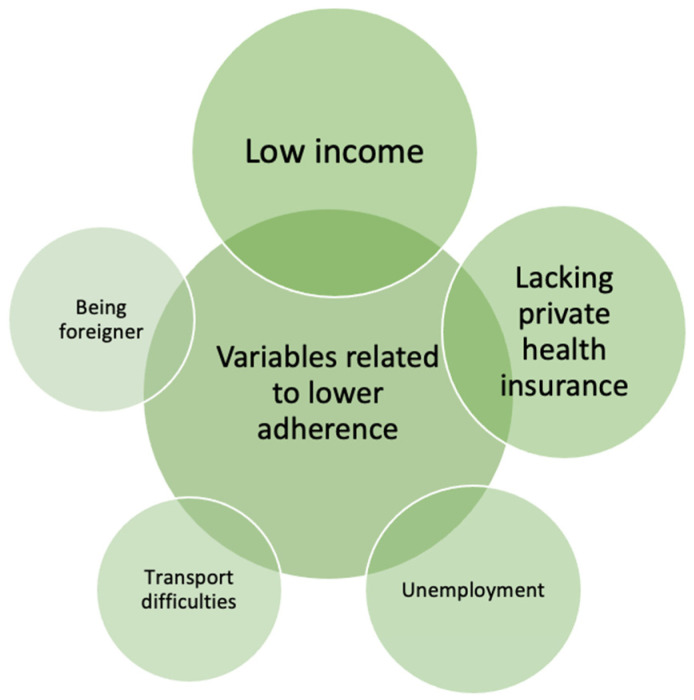
Variables associated with low socioeconomic level and lower adherence.

Low income and a lack of socioeconomic resources [16,18,19,23,24,27] appear as common barriers to adherence in six of the researched studies, which underlines the weight of this factor for adherence to a breast cancer–screening program. Figure 2 shows the main variables related to socioeconomic level that influence adherence to a breast cancer–screening program.

Low income is the variable that is present in most of the studies analyzed, and it is closely related to the lack of private health insurance and to unemployment (Figure 2). Participants with a higher income level showed a 7.1% higher participation rate (relative risk—RR: 1.71, *p* < 0.001) [27]. Furthermore, when comparing low-income women with those of moderate (OR: 0.69) or high income (OR: 0.85), the latter two are less likely to report barriers to participation in this screening [18]. Another barrier reported by women was the transportation difficulties they experience to reach their corresponding health service facility, where women who have this as a barrier are 26.4% less likely to attend the screening [21].

Another factor common among several of the articles analyzed [21,25,30] is the lack of private health insurance. Having medical insurance (*p* = 0.0025) is related to greater adherence to mammography [25], a probability three times higher [21] than those women who do not have it, and it is also a factor that favors women’s reporting fewer barriers to adherence to a screening program when they are asked about it [24].

### 3.4. Family and Individual Factors

Having a family or personal history of cancer and being married or of older age become protective factors for adherence in women. Having a high level of education; having good local language speaking ability; and knowing about the program are all associated with higher levels of adherence, as shown in Table 3.

Having a personal and/or family history of cancer has been related to better knowledge and awareness of this health problem and with early detection of future relapses [25,30,31]. Being married or in a couple has also been related to higher adherence to these programs [17,25,26,30]. An et al. [26] observed that being married is related to a higher level of awareness of breast cancer–screening programs (OR: 29.152, *p* < 0.01). Shon et al. [17] concluded that the odds of never having undergone mammography were higher in unmarried women (OR: 1.74, 95% CI [1.08–2.82]). Older age has also been associated with higher participation rates [31].

However, the lack of health education and low educational level [15,19,22,25,27,31] are risk factors for adherence to the screening program. In contrast, high educational level and knowledge of the screening are strongly associated with higher participation. Lee et al. [31] highlight that women with a school diploma (OR: 13.203, *p* < 0.01) or academic degree (OR: 6.750, *p* < 0.01) showed higher levels of mammography use, as did those who had heard of the screening program (OR: 36.250, *p* < 0.01). Finally, having a good English-speaking ability [25] is a determinant factor for better adherence (*p* = 0.0021).

### 3.5. Health Information Gap and Medical Mistrust

Table 4 shows the main factors related to the levels of information about breast cancer screening between women and their confidence and involvement in healthcare systems.

A study including 159 ethnically diverse women showed that after educational sessions, a decrease of 5% (*p* = 0.04) in the lack of knowledge about breast screening was observed [22] (Table 3). Moreover, there was an improvement in the ability to identify the correct starting age for screening after the session (14.8% presession vs. 37.7% postsession) and the frequency of screening (39.3% vs. 90.2). Another practice that seems to be associated with greater participation in screening programs is having an annual health checkup [25,26,28] (Table 4). According to An et al. [26], women are 16 times more likely to participate in the screening program if they undergo this health control (OR: 16.148, *p* < 0.05). Orji et al. conclude that women who had received an annual health checkup were more likely to participate in breast cancer–screening programs (OR: 5.86, *p* < 0.05) compared with those who did not receive it [28].

It is demonstrated that a good doctor–patient relationship helps to increase levels of adherence [30] (OR: 1.485), which encourages healthcare providers to recommend patients participate in screening programs (*p* = 0.0027) [25]. In a study carried out in migrant women living in the US, longer length of stay (*p* < 0.001) and acculturation (*p* = 0.002) have been related to higher levels of adherence [29]. They also showed that higher levels of medical distrust were associated with lower participation in screening [29], although this factor was found to be not significantly associated with greater participation in another study [24]. However, acculturation also seems to be a protective factor according to the study by An et al. [26].

## 4. Discussion

As Krieger demonstrates, the exacerbation of inequities during the COVID-19 pandemic owing to structural racism in the US [32]; these results agree with the existing cultural barriers due to racism and injustices in healthcare systems across the world today. Johnson-Agbakwu et al. showed the inequities in mortality between white and Black populations [33] and how these have been exacerbated by the recent COVID-19 pandemic, seeking to propose measures to resolve this situation. Also in Italy, a study on the epidemiological characteristics of COVID-19 cases in non-Italian nationals confirmed that compared with Italians, undocumented foreigners have a greater risk of severe clinical outcomes [34]. Along the same lines, Ponce-Blandón et al. analyzed the complex reality faced by migrants who cross the Strait of Gibraltar for a better life [35], identifying various cultural barriers encountered by health professionals who were unable to provide culturally appropriate help when they were caring for people of different races/ethnicities: factors such as language, religious beliefs, cultural habits and prejudices, among others [36]. These factors could be the reason for lower participation in and a greater lack of knowledge of population-based health and early-detection programs. Molina-Barceló et al. [37] identified the profile of women who participated to a lesser extent in breast cancer screening in Spain: young, migrant and/or of nonwhite race and/or ethnicity.

The results on low incomes are consistent with other research that has studied barriers to accessing health services in general, not only in relation to adherence to screening programs. Zhou et al. [38] and Shahar et al. [39] studied Chinese and Malaysian populations and demonstrated a direct relationship between health variables and economic resources, which are interrelated, creating the so-called Hortwiz circle [40]: poverty–unhealthy–low income–poverty, suggesting that public health strategies should be directed at vulnerable populations. Lund et al. [41] found a significant relationship between poverty levels and mental illnesses owing to social exclusion, stress and barriers to accessing healthcare, demonstrating the influence of a lack of economic resources on physical, mental and social health across the world. Some studies show that a large population in the US are still without health insurance and that this population tends to be those with fewer resources [42]. Despite having private health insurance, the barriers to accessing basic health programs continue to be greater among those with fewer resources [43]. According to Serral et al. [44], the Spanish women who had higher participation rates in breast cancer screening were those (i) between 60 and 69 years, (ii) with high incomes, (iii) with private health insurance and (iv) born in a country with a Human Development Index (HDI) score over 0.8, which supports the results obtained in our study.

We have also demonstrated the importance of educating the population and informing women about breast cancer–screening programs. In fact, other studies have highlighted the importance of implementing health education programs in schools, with the aim of training children to understand health information from an early age [45]. Other studies have also shown the multiple positive effects of the implementation of annual health checkups and strongly recommend them to improve lifestyle habits [46]. Although population screening programs are intended to promote health equity, it cannot be denied that there are still inequities, with a tendency to lower participation in those social groups who are vulnerable, determined by all the barriers discussed above. For example, the participation rates in cancer screenings by age in the US, where 88% of the articles in our research come from, do not meet the required standards [47]. Therefore, boosting adherence in these vulnerable populations is a necessary global action.

Given the main barriers encountered in this study, some measures are proposed that aim to improve screening coverage in vulnerable populations. The implementation of awareness programs for migrant and/or nonwhite women could be useful to raise their awareness of the importance of breast cancer screening. To be accessible to them, the campaigns could be implemented in places related to their everyday lives, such as schools, the media and/or supermarkets. An example of this was conducted by Alkhasawneh et al. [48], who implemented a breast cancer education program for Arab women to increase knowledge of the subject and participation in screenings, and the results were clearly positive. Serral et al. [44] invited us to inform the population, focusing on women from the most vulnerable groups, of the benefits and risks of participating in a breast cancer–screening program, so that women can make informed decisions.

Moreover, work should be conducted on the implementation of annual health checkups, with the intention of recruiting women who are susceptible to neglecting screening. In this regard, we should work with health professionals because they are the key actors to enrolling patients who meet the requirements—informing and reminding patients of the importance of participating in screening programs. In addition, efforts should be aimed to avoid the health information gap and mistrust in healthcare systems in order to overcome the inequities that exacerbate the lack of adherence. Ponce-Blandón et al. [35] identified some difficulties to managing cultural differences from healthcare workers’ perspectives and proposed measures to educate these professionals on the values of diversity and respect. It is crucial to guarantee medical trust in healthcare professionals and in healthcare systems in order to improve doctor–patient relationships, increasing satisfaction and the adherence to screening, with a particular focus on the diversity and cultural integration of all people.

Most of the studies were descriptive observational studies (95%), which gives a low level of evidence to the results; only two experimental studies were included (5%). However, given that the objective of this research was not to evaluate a health intervention but rather to analyze the factors and barriers that influence the lack of adherence to breast cancer–screening programs, we expected to find more descriptive studies than other types. Another limitation was that most of the publications analyzed were carried out in the US (88%); only two of the 17 were conducted outside of the US, one in Asia and one in Australia. For this reason, we encourage European and African researchers, as well as professionals across the world, to focus their research on this vital topic to gain health equity, which is included as an objective in the 2030 Agenda for Sustainable Development (Goal 3: ensure healthy lives and promote well-being for all at all ages).

## 5. Conclusions

**Scheme 2:**
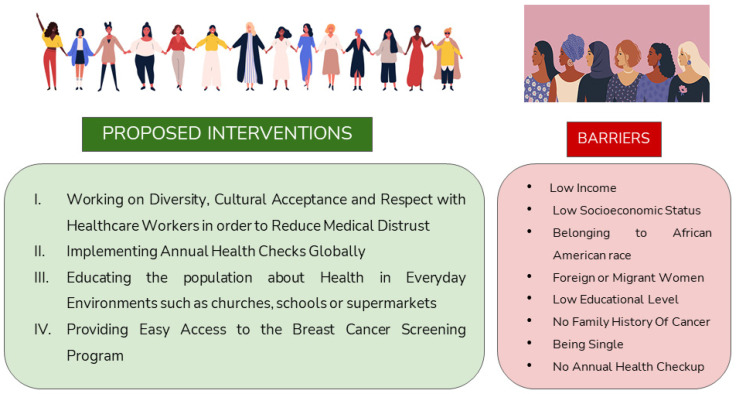
Proposed interventions and main barriers detected for breast cancer–screening program.

Multiple barriers have been found to affect adherence to breast cancer–screening programs. The target populations with the lowest adherence in our study were Black, Asian, Hispanic and foreign women. Those with barriers are detailed in Scheme 2. A lack of knowledge of these screening programs and medical mistrust in healthcare systems owing to cultural differences also exacerbate this health issue. In general, the adoption and dissemination of breast cancer–screening programs is still deficient in a large part of vulnerable populations, where the influential barriers are associated with race and/or ethnicity (47% of the cases) and low socioeconomic level (35.3%). However, we observed important favorable changes in those cases in which the population undergoes health educational sessions, is informed about the screening or is recommended by health professionals to attend. Therefore, we propose interventions to avoid these social disparities and avoid the low levels of adherence in vulnerable populations, which are described in Scheme 2. We should mention that more in-depth studies on this health problem will be very useful to continue improving participation in and relieving the difficulties hindering women’s adherence to these early-detection programs.

## Figures and Tables

**Table 1:** Main results of the 17 studies included in the review.

Authors/Year/Country ^1^	Journal	Title	Main Results
Haas et al. [15]/2016/USA	Cancer	Disparities in the Use of Screening Magnetic Resonance Imaging (MRI) of the Breast in Community Practice by Race, Ethnicity, and Socioeconomic Status	Among patients with a lower risk of developing breast cancer (<20%), non-Hispanic white women were 62% more likely than nonwhite women to undergo MRI (95% CI [1.32–1.98]). Of these, those with higher levels of education (43%), and especially those with academic degrees (132%), were more likely to receive MRI.
Elewonibi et al. [16]/2018/USA	Journal of Immigrant and Minority Health	Examining Mammography Use by Breast Cancer Risk, Race, Nativity, and Socioeconomic Status	Being a foreigner is an independent factor that decreases the probability of receiving a mammogram by about 10% (OR: 0.904, *p* < 0.05). If the variables of socioeconomic status and social resources are added as controls, higher levels are related to higher probability of mammography.
Shon et al. [17]/2019/USA	PLoS ONE	Predictors of never having a mammogram among Chinese, Vietnamese, and Korean immigrant women in the U.S.	The odds of never having had a mammogram were higher in Korean women (OR: 1.80, 95% CI [1–3.22]), unmarried women (OR: 1.74, 95% CI [1.08–2.82]) and non-US citizens (OR 2.56, 95% CI [1.44–4.55]). Conversely, they were lower in women aged 50–59 (OR:0.37, 95% CI [0.15–0.89]) and 60–69 years (OR: 0.36, 95% CI [0.17–0.75]) compared with those aged 70–85 years.
Henderson et al. [18]/2020/USA	Journal of Women’s Health	The Role of Social Determinants of Health in Self-Reported Access to Health Care Among Women Undergoing Screening Mammography	Compared with white women, more barriers were reported among Black (OR: 1.30) and Hispanic (OR:1.66) women. They were also higher in areas with a high diversity index (OR: 1.28), where they were lower among women with moderate (OR: 0.69) or high incomes (OR: 0.85).
Lee et al. [19]/2021/USA	JAMA Network Open	Comparative Access to and Use of Digital Breast Tomosynthesis (DBT) Screening by Women’s Race/Ethnicity and Socioeconomic Status	Black women experienced lower DBT use (RR: 0.83) compared with white women (RR: 0.98); also, women with lower educational attainment (RR: 0.79–0.88) and lower income (RR 0.89) had lower use relative to educated women (RR: 0.90–0.96) and those with the highest income (RR: 0.99).
Warnecke et al. [20]/2021/USA	Cancer Epidemiology, Biomarkers and Prevention	Multilevel Examination of Health Disparity: The Role of Policy Implementation in Neighbourhood Context, in Patient Resources, and in Healthcare Facilities on Later Stage of Breast Cancer Diagnosis	After adjusting for setting, mode of detection and resources, no significant differences were found in the late diagnosis of breast cancer among non-Hispanic Black and Hispanic women compared with non-Hispanic white women.
Gibbons J. [21]/2021/USA	Race and Social Problems	Neighbourhood Racial/Ethnic Composition and Medical Discrimination’s Relation to Mammograms: A Philadelphia Case Study	Black and Hispanic women were 5 times more likely to have experienced discrimination (11.3% and 11.2%, respectively) than white women (2.2%). Those who had access problems owing to transportation were 26.4% less likely to attend a screening, while those with private insurance were 323.2% more likely.
Cullerton et al. [22]/2016/Australia	Health Promotion Journal of Australia	Cancer screening education: can it change knowledge and attitudes among culturally and linguistically diverse communities in Queensland, Australia?	After education sessions, a decrease of 5% (*p* = 0.04) in the lack of knowledge about breast screening was observed. Likewise, after the session, there was an improvement in the ability to identify the correct age to start screening (14.8% presession vs. 37.7% postsession) and the frequency of screening (39.3% vs. 90.2).
Molina et al. [23]/2017/USA	Journal of Racial and Ethnic Health Disparities	Neighbourhood predictors of mammography barriers among US-based Latinas	The proportion of women who reported sociocultural, economic and lack of knowledge reasons for not having undergone mammography were 0.19, 0.31 and 0.35, respectively. Women residing in areas with a lower concentration of Latinos less frequently reported economic and lack of knowledge reasons (*p* < 0.05).
Kim et al. [24]/2018/USA	Cancer	Gendered and Racialized Social Expectations, Barriers, and Delayed Breast Cancer Diagnosis	Women who identified barriers were more likely to receive follow-up mammography screening (31.2% vs. 48.4%, *p* < 0.01). Black women (prediction value 1.91, *p* < 0.01), those exhibiting distrust (prediction value 0.92, *p* < 0.01) and those living in poverty (prediction value 4.69, *p* < 0.005) were less likely to report barriers.
Jin et al. [25]/2019/USA	Ethnicity and Disease	Analyzing Factors of Breast Cancer Screening Adherence among Korean American Women Using Andersen’s Behavioral Model of Healthcare Services Utilization	Personal history of cancer (*p* = 0.0027), having undergone an annual health checkup (*p* < 0.0001), having health insurance (*p* = 0.0025), receiving a recommendation by healthcare personnel (*p* = 0.0027) and high level of English (*p* = 0.0021) are related to higher adherence to mammography.
Lee et al. [19]/2019/USA	Journal of Evidence-Based Social Work	Andersen’s Behavioral Model to Identify Correlates of Breast Cancer Screening Behaviors among Indigenous Women	Older women (OR: 1.116, *p* < 0.001), those with a family history of cancer (OR: 2.742, *p* < 0.05) and those with a school diploma (OR: 13.203, *p* < 0.01) or academic degree (OR: 6.750, *p* < 0.01) showed higher levels of mammography use. Those who had heard of the screening program also showed higher levels (OR: 36.250, *p* < 0.01).
An et al. [26]/2020/USA	Journal of Immigrant and Minority Health	Literacy of Breast Cancer and Screening Guideline in an Immigrant Group: Importance of Health Accessibility	Marital status of married (OR: 29.152, *p* < 0.01) and having undergone an annual health checkup (OR: 16.148, *p* < 0.05) is related to a higher level of awareness of breast cancer–screening programs.
Chan et al. [27]/2021/Singapore	Frontiers in Oncology	Cancer Screening Knowledge and Behavior in a Multi-Ethnic Asian Population: The Singapore Community Health Study	Of the participants, only 35.1% claimed to have participated in a breast cancer–screening program. Participants with higher educational levels showed a 22% higher participation rate (aPR: 1.22, *p* < 0.032), as did those with high income by 7.1% (aPR: 1.71, *p* < 0.001).
Orji et al. [28]/2021/USA	Cancer Causes and Control	Racial disparities in routine health checkup and adherence to cancer screening guidelines among women in the United States of America	Women who had received an annual health examination were more likely to participate in cervical (OR: 3.24, *p* < 0.05) and breast (OR: 5.86, *p* < 0.05) cancer–screening programs, compared with those who did not receive screening—with the exception of Hispanic women, in whom this relationship was not observed.
Hong et al. [29]/2018/USA	BMC Women’s Health	Factors affecting trust in healthcare among middle-aged to older Korean American women	A longer stay in the US increases the level of trust in the health system (*p* < 0.001). The feeling of discrimination causes distrust in the health system (*p* < 0.001). Acculturation was related to a higher level of trust in healthcare providers (*p* = 0.002).
Agrawal et al. [30]/2021/USA	Int. Journal of Environmental Research and Public Health	Factors Associated with Breast Cancer Screening Adherence among Church-Going African American Women	Older age (OR: 1.015), having health insurance (OR: 2.388), having a good doctor–patient relationship (OR: 1.485) and having a previous diagnosis of cancer (OR: 2.244) were associated with a higher level of adherence.

^1^ The studies are listed organized by year of publication and main focused topic: (i) race and/or ethnicity and/or foreign nationality (the first 6), (ii) low educational level (the middle 8) and (iii) medical mistrust (the last 2).

**Table 2:** Barriers to breast cancer–screening adherence.

Barriers to Adherence	*n* ^1^	% ^2^
Race and/or ethnicity and/or foreign nationality	8	47
Low socioeconomic level and lack of resources	6	35.3
Low educational level	5	29.4
No family history and being single	5	29.4
Health information gap and medical mistrust	4	23.5
Failure to attend annual health checkups	3	17.6
Lack of private health insurance	3	17.6

^1^*n* = number of studies on the research that include this barrier; ^2^ % = percentage of the total number of studies on the research (*n* = 17).

**Table 3:** Adherence to breast cancer–screening program according to family/personal history, marital status and educational level.

Authors/Year/Country	MS ^1^	PC ^2^	Age	HE ^3^	ES ^4^	KP ^5^
Agrawal et al./2021/USA [30]	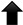	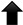	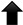			
An et al./2020/USA [26]	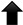					
Jin et al./2019/USA [25]	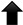	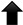		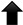	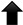	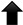
Lee et al./2019/USA [31]		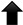	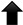	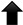		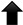
Shon et al./2019/USA [17]	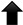		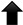			
Lee et al./2021/USA [19]				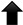		
Chan et al./2021/Singapore [27]				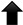		
Haas et al./2016/USA [15]				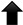		

^1^ MS: marital status, married or in a couple; ^2^ PC: previous diagnosis of cancer; ^3^ HE: high level of education; ^4^ ES: good English-speaking ability; ^5^ KP: knowledge of the program. 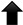 = Higher adherence.

**Table 4:** Adherence to breast cancer–screening program according to information, confidence and involvement in health.

Authors/Year/Country	RHC ^1^	EBCS ^2^	MM ^3^	A ^4^	AHC ^5^
Agrawal et al./2021/USA [30]	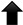				
Orji et al./2021/USA [28]					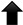
An et al./2020/USA [26]				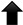	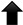
Jin et al./2019/USA [25]	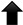				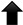
Kim et al./2018/USA [24]					
Hong et al./2018/USA [29]				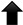	
Cullerton et al./2016/Australia [22]		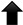			

^1^ RHC: favorable relationship with the health care provider; ^2^ EBCS: education about breast cancer screening; ^3^ MM: medical mistrust; ^4^ A: acculturation; ^5^ AHC: annual health checkup. 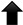 = Higher adherence.
